# Proceedings From the First International Workshop at Sidra Medicine: “Engineered Immune Cells in Cancer Immunotherapy (EICCI): From Discovery to Off-the-Shelf Development”, 15^th^–16^th^ February 2019, Doha, Qatar

**DOI:** 10.3389/fimmu.2020.589381

**Published:** 2021-01-14

**Authors:** Bella Guerrouahen, Muhammad Elnaggar, Anjud Al-Mohannadi, Dhanya Kizhakayil, Chiara Bonini, Reuben Benjamin, Renier Brentjens, Christian J. Buchholz, Giulia Casorati, Soldano Ferrone, Frederick L. Locke, Francisco Martin, Axel Schambach, Cameron Turtle, Paul Veys, Hans J. van der Vliet, Cristina Maccalli, The EICCI Faculty Group

**Affiliations:** ^1^ Research Department, Sidra Medicine, Doha, Qatar; ^2^ Experimental Hematology Unit, University Vita-Salute San Raffaele and Hospital San Raffaele Scientific Institute, Milan, Italy; ^3^ Division of Cancer Studies, King’s College Hospital, London, United Kingdom; ^4^ Cellular Therapeutics, Department of Medicine, Memorial Sloan Kettering Cancer Center, New York, NY, United States; ^5^ Research Unit for Molecular Biotechnology and Gene Therapy, Paul-Ehrlich-Institut, Langen, Germany; ^6^ Experimental Immunology Unit, University Vita-Salute San Raffaele and Hospital San Raffaele Scientific Institute, Milan, Italy; ^7^ Department of Surgery, Massachusetts General Hospital, Harvard Medical School, Boston, MA, United States; ^8^ Department of Blood and Marrow Transplant and Cellular Immunotherapy, Moffitt Cancer Center, Tampa, FL, United States; ^9^ Pfizer/University of Granada/Andalusian Regional Government, Genomic Medicine Department, Granada, Spain; ^10^ Institute of Experimental Hematology, Hannover Medical School, Hannover, Germany; ^11^ Division of Hematology/Oncology, Boston Children’s Hospital, Harvard Medical School, Boson, MA, United States; ^12^ Clinical Research Division, Fred Hutchinson Cancer Center, Seattle, WA, United States; ^13^ Bone Marrow Transplant Unit, Great Ormond Street (GOS) Hospital, and University College London GOS Institute of Child Health, London, United Kingdom; ^14^ Hans van Der Vliet, Department of Medical Oncology, Amsterdam UMC, VU University and Cancer Center, Amsterdam, Netherlands; ^15^ Lava Therapeutics, Utrecht, Netherlands

**Keywords:** cancer, immunotherapy, CAR-T cells, TCR engineered lymphocytes, CAR-NK cells, monoclonal antibody, clinical trial, off-the-shelf development

## Abstract

The progress in the isolation and characterization of tumor antigen (TA)-specific T lymphocytes and in the genetic modification of immune cells allowed the clinical development of adoptive cell therapy (ACT). Several clinical studies highlighted the striking clinical activity of T cells engineered to express either Chimeric Antigen (CAR) or T Cell (TCR) Receptors to target molecularly defined antigens expressed on tumor cells. The breakthrough of immunotherapy is represented by the approval of CAR-T cells specific for advanced or refractory CD19^+^ B cell malignancies by both the Food and Drug Administration (FDA) and the European Medicinal Agency (EMA). Moreover, advances in the manufacturing and gene editing of engineered immune cells contributed to the selection of drug products with desired phenotype, refined specificity and decreased toxicity. An important step toward the optimization of CAR-T cell therapy is the development of “off-the shelf” T cell products that allow to reduce the complexity and the costs of the manufacturing and to render these drugs available for a broad number of cancer patients. The Engineered Immune Cells in Cancer Immunotherapy (EICCI) workshop hosted in Doha, Qatar, renowned experts, from both academia and industry, to present and discuss the progress on both pre-clinical and clinical development of genetically modified immune cells, including advances in the “off-the-shelf” manufacturing. These experts have addressed also organizational needs and hurdles for the clinical grade production and application of these biological drugs.

## Introduction

Cancer immunotherapy is aimed at a driving patient’s immune system to attack tumor cells. The great advances achieved in this field during the last two decades, lead to the emerging role of immunotherapy as the “fifth pillar” of cancer treatment, together with surgery, chemotherapy, radiotherapy and targeted therapy (1).

Adoptive cell therapy (ACT) with tumor antigen (TA)-specific T lymphocytes has been clinically developed at an unparalleled pace (2–4). In particular, the approach of the genetic engineering of T or NK cells to target and destroy cancer cells, revealed as powerful and, in some cases, unprecedented in term of clinical success for the treatment of patients with aggressive malignancies, refractory to other therapeutic interventions (5–7).

T cells engineered with chimeric antigen receptors (CARs), that combine the antigen binding region of antibodies and T cell signaling domains responsible for activation (8), represented the breakthrough of cell-based immunotherapy (1, 2, 3). Different CARs have been engineered to target a variety of antigens expressed by either hematologic or solid tumors, that are listed by Sadelain and colleagues (9). The initial clinical application of CAR-T cells was quite disappointing in terms of patients’ responses, due to the inefficient expansion and persistence of CAR-T cells *in vivo* (10–12). Additional modifications of the structure of CARs, by including co-stimulatory domains allowed the achievement of clinical benefit through the treatment of patients with B cell malignancies overexpressing CD19 (13–18). Strikingly response rates in the range of 57%–82%, with complete response rate of 52-60%, were detected upon the infusion of CD19-CAR-T cells in patients with B cell malignancies refractory to prior treatments (7, 8, 9). These results led to the accelerated approval by both FDA and EMA of two drug products: 1. tisagenlecleucel/Kymriah for the treatment of children and young adult with acute lymphoblastic leukemia (ALL) (13, 14, 19–21), and for the treatment of adults with relapsed/refractory Diffuse Large B cell lymphoma (DLBCL) (22).

Axicabtagene Ciloleucel/Yescarta for the Treatment of Adult Patients With Relapsed/Refractory Non-Hodgkin Lymphoma (NHL), including **Table 1** (14, 16, 18).

**Table 1:** Summary of principal clinical studies of CAR-T/NK cells.

Trial registration N.	Sponsor	Country	Agent/ cell source and type	Indication	Note
NCT03601442	Novartis Pharmaceuticals	US	LV CAR-T (CTL019)	B-ALL/B cell lymphomas	Available in the market (Kymriah)
NCT02348216	Kite Pharma	US	RV CAR-T (KTE-C19, Axi-cel)	B-ALL/DLBCL	Available in the market (Yescarta)
NCT02601313	Kite Pharma	US	2^nd^ generation -Autologous anti-CD19 CAR-T cell	Mantle Cell Lymphoma	Available in the market (Tecartus)
NCT01044069	Kite Pharma	US	2^nd^ generation -Autologous anti-CD19 CAR-T cell	CLL,	Phase 1/2
NCT01593696	NCI	US	2^nd^ generation -autologous anti-CD19 CAR-T cell	NHL, ALL, CLL	Phase 1/2
NCT00586391	Baylor College of Medicine, Texas Children’s Hospital	US	2^nd^ generation -Autologous anti-CD19 CAR-T cell	Non-Hodgkin’s lymphoma, ALL, CLL	Phase 1/2
NCT00924326	NCI	US	Anti-(CD19)-CAR PBL	Primary Mediastinal B-cell Lymphoma, Diffuse Large B-cell Lymphoma, mantle cell	Phase 1/2
NCT01626495	Children’s Hospital of Philadelphia, University of Pennsylvania	US	2^nd^ generation -Autologous anti-CD19 CAR-T cell	B-ALL, CD19+ Leukemia and Lymphoma	Phase1/2
NCT03056339	M.D. Anderson Cancer Center	US	Allogeneic CD19-CD28-zeta-2A-iCasp9-IL15-transduced cord blood-NK cells	Refractory B cell Malignancies	Phase 1
NCT02808442	Institut de Recherches Internationales Servier	France, Japan, UK and US	Allogeneic UCAR19	Pediatric patients with relapsed or refractory CD19^+^ B-ALL	Phase 1
NCT02208362	City of Hope Medical Center	US	Autologous anti-IL13Rα2 CAR-T cell	Glioblastoma	Phase 1
NCT00902044	Baylor College of Medicine	US	Autologous HER2-CAR-T cells	Sarcoma	Phase 1
NCT02107963	NCI	US	3rd generation Autologous anti-GD2-CAR-T cells	Sarcoma, Osteosarcoma, Neuroblastoma, Melanoma	Phase 1
NCT04097301	MolMed	Italy	Autologous CD44v6 CAR T-cells- HSV-TK Mut2 gene	Multiple Myeloma	Phase 1/2
–	National Cancer Institute	US	(EGFRv)III Chimeric antigen receptor (CAR) transduced PBL	Malignant Gliomas Expressing EGFRvIII (Astrocytoma, Glioblastoma, Glioma, Gliosarcoma, Brain Neoplasms)	Phase 1/2
–	Glycostem Therapeutics		Allogeneic cord blood-derived CD19-NK cells (oNKord®)	Elderly AML	Phase 1
NCT03294954	Baylor College of Medicine	US	2^nd^ generation -anti-GD2-IL15^+^CAR-NKT cells	Neuroblastoma	Phase 1

AML, Acute Myeloid Leukemia; BCMA, B-Cell Maturation Antigen; CD44v6, CD44 Variant 6; GD2, Disialoganglioside; HSV-TK Mut2, Mutated Herpes Simplex Virus Thymidine Kinase; IL13Rα2, Interleukin 13 Receptor Subunit Alpha 2; LV, Lentiviral Vectors; NCI, National Cancer Institute; NKG2D, C-Type Lectin-Like Receptor RV, Retroviral Vectors; UK, United Kingdom; US, United States; ALL, Acute lymphoblastic leukemia; CLL, Chronic Lymphoblastic Leukemia.

More recently, a third product, brexucabtagene autolucel (Tecartus) has been approved for the treatment of relapsed or refractory mantle cell lymphoma. This approval was granted based on the results of the ZUMA-2 (NCT02601313) clinical trial, showing 87% of ORR, with a complete remission (CR) rate of 62% (23).

CAR-T cells represent promising therapeutic options also for solid tumors, although they are still under clinical development and do not yet have proven their clinical efficacy (**Table 1**) (24). The principal limitations for their clinical activity are: i. paucity of tumor specific antigens and ii. the low efficiency of T cells in penetrating the tumor microenvironment and homing to the tumor site, and iii. their limited functional activity within the tumor (24).

In addition, efforts are ongoing at different leading groups to develop allogeneic CAR-T cell therapies, in order to simplify the manufacturing process, to reduce the costs and rendering these drugs available to larger cohorts of patients (**Table 1**).

Promising results have been obtained also for the alternative approach of engineering T lymphocytes with TA-specific T Cell Receptor (TCR) for the treatment of solid tumors and multiple myeloma, including patients with advanced metastatic malignancies (**Table 2**) (2, 4, 25–31). This methodology has been applied for the generation of high avidity tumor-specific T cells. The choice of the targeted antigens is critical for the efficacy of this type of therapy and to prevent off-target reactivity (32, 33). The targeting of overexpressed antigens and neo-antigens, that are not shared with non-embryonic normal tissues, can lead to the cell-mediated killing of tumor cells without severe toxicities due to the targeting of normal tissue(s) (27–29, 34, 35).

**Table 2:** Summary of principal clinical studies of TCR-engineered T cells.

Trial registration	Sponsor	Country	Agent/ cell source and type	Indication	Note
NCT01967823	NCI	US	Anti-NY-ESO-1 mTCR	NY-ESO-1 Expressing Cancers	Phase 2
NCT02111850	NCI	US	Anti-MAGE-A3-DP4 TCR	Various solid metastatic tumors (Targeting MAGE-A3) in HLA-DP04 positive patients	Phase 1/2
NCT02408016	Fred Hutchinson Cancer Research Center/NCI	US	WT1-TCRc4 Tcm/Tn Lymphocytes	Non-small Cell Lung Cancer, Mesothelioma	Phase 1/2
NCT01621724	Cell Medica Ltd/University College, London/Cell Therapy Catapult	UK	WT1 TCR-	Leukaemia (AML CML)	Phase 1/2
NCT03250325	GlaxoSmithKline/Adaptimmune	US	NY-ESO-1 TCR	Synovial Sarcoma	Phase 1/2
–	GlaxoSmithKline/Adaptimmune	US	NY-ESO-1 TCR	Non-small Cell Lung Cancer	Phase 1/2
–	GlaxoSmithKline/Adaptimmune	US	NY-ESO-1	Ovarian cancer	Phase 1/2
NCT03139370	Kite Pharma	US	KITE-718 (MAGE-A3/A6) TCR	MAGE-A3/A6-positive solid tumors	Phase 1/2
NCT03399448	Parker institute for Cancer Immunotherapy/ University of Pennsylvania	US	NY-ESO-1 TCR	Multiple myeloma, Sarcoma, Melanoma	Phase 1/2
NCT02840110	Unum Therapeutics Inc.	US	ACTR TCR	Multiple Myeloma	Phase 1/2
NCT02743611	Bellicum Pharmaceuticals	US	BPX-701/PRAME-TCR	AML, MDS, metastatic Uveal melanoma	Phase 1/2
NCT03247309	Immatics Biotechnologies (Tuebingen, Germany)/M.D. Anderson Cancer Center	US	IMA-201	Various solid tumors (Head and Neck Squamous Cell Carcinoma, Non-small Cell Lung Cancer)	Phase 1
NCT03912831	NCI/Kite Pharma	US	KITE-439	HPV16+ Tumors (relapsed or refractory)	Phase 1
–	Fred Hutchinson/Juno	US	Anti-mesothelin TCR	Pancreatic cancer	
NCT01567891	Adaptimmune	US	Enhanced TCRs Specific for NY-ESO-1	Refractory Ovarian Cancer	Phase 1/2
NCT03412877	NCI	US	Autologous T-Cells Genetically Engineered to Express T-Cell Receptors Reactive Against Mutated Neoantigens in People With Metastatic Cancer	Glioblastoma Non-Small Cell Lung Cancer Ovarian Cancer Breast Cancer Gastrointestinal/Genitourinary Cancer	Phase 2
NCT03907852	TCR^2^ Therapeutics	US	Autologous genetically engineered T cells expressing a single-domain antibody that recognizes human Mesothelin (TC-210 T cells)	Mesothelioma Cholangiocarcinoma Recurrent Ovarian Cancer Non Small Cell Lung Cancer Non Small Cell Lung Cancer Metastatic	Phase 1/2
NCT04323657	TCR^2^ Therapeutics	US	Autologous genetically engineered T cells expressing a single-domain antibody that recognizes human CD19 (TC-110 T cels)	Non Hodgkin Lymphoma ALL DLBCL Primary Mediastinal Large B Cell Lymphoma CML FL	Phase 1/2

HPV, Human Papilloma Virus; EGFRVIII, Epidermal Growth Factor Receptor Variant III; NY-ESO-1, New York Esophageal Squamous Cell Carcinoma 1; PRAME, Melanoma Antigen Preferentially Expressed in Tumors; MAGE, Melanoma Antigen; WT1, Wilms Tumor 1; NCI, National Cancer Institute; US, United States; AML, Acute Myeloid Leukemia; CML, Chronic Myelogenous Leukemia; DLBCL, Diffuse Large B Cell Lymphoma; FL, Follicular Lymphoma.

Gene editing through the targeted knock out of genes or the insertion of suicide genes represents an innovative strategy that has been applied to tumor-specific engineered immune cells with the aims of either increasing their specificity and *in vivo* persistence or decreasing the induction of possible toxicities or allogeneic rejection, respectively (36–39). The application of this technique allowed also to generate universal/”off-the-shelf” CAR-T cells using the peripheral blood of healthy volunteers as source of immune cells (40–47). This strategy is currently under clinical development with few clinical trials ongoing in EU and USA (42, 44, 48).

All the topics mentioned above have been addressed in a comprehensive manner in the context of the first international workshop in Doha “Engineered immune cells in cancer immunotherapy: from discovery to off-the-shelf development” (15^th^–16^th^ February 2019, Doha, Qatar). Renowned speakers from both academia and industry who pioneered the field gathered in Doha bringing high level discussions on scientific and clinical advances and bringing the participants at the forefront of this rapidly evolving topic. A satellite mini-symposium at the initial opening of the workshop, through its educational contents, has provided basic knowledge of cancer immunology, immunotherapy and cell-based therapies to health care practitioners, researchers and students. Poster sessions have also offered the opportunity for active involvement of young researchers and under-graduate students. This report summarizes key data and highlights from each session.

## The Clinical Evolution of CAR-T Cell Therapy

During the past few years, CAR-T cells, either CD28/CD3ζ (4, 5) or 4-1BB/CD3ζ CARs (6), targeting CD19^+^ B cell malignancies have demonstrated safety and clinical activity in the context of multiple Phase I/II clinical trials (49). These products have been used for the treatment of either pediatric and adult patients with relapsed or refractory ALL, showing high CR rate (50–55). Similarly, CD19-CAR-T cells showed impressive clinical activity in relapsed/refractory pediatric-adolescent or adult Non-Hodgkin Lymphoma (NHL) with 40-63% of CR (49, 56–59). These unprecedented results lead to the rapid approval by both FDA and EMA and the commercialization of these Advanced Therapeutic Medicinal Products (ATMPs) (60, 61).

Associate Prof. Cameron Turtle (Fred Hutchinson Cancer Research Center, USA) kicked off the first session of the workshop with a keynote lecture summarizing the aforementioned results and focusing on the factors impacting the response to CD19 targeting CAR-T cell immunotherapy in adults with ALL and NHL in a clinical trial at Fred Hutchinson Cancer Research Center, Seattle, WA. Different factors were found to affect the patients’ clinical responses, including the dose of infusion of CAR–T cells, their cellular expansion and persistence *in vivo*, the tumor burden, and the conditioning regimen used for lymphodepletion (50, 60). Low levels of circulating CAR-T cells were identified as one of the mechanisms of resistance to CAR-T cell therapies. In some ALL patients, antigen loss was observed in tumor cells following the treatment with CD19-CAR-T cells, as a consequence of either missense mutations or alternative splicing of the CD19 encoding gene (62–64). In ALL patients low levels of lactate dehydrogenase (LDH) and normal platelet counts in the blood prior to lymphodepletion followed by the treatment with cyclophosphamide/fludarabine (Cy/Flu) compared to Cy regimen alone were identified as factors associated with better disease-free survival (DFS) (60). Furthermore, DFS was improved in ALL patients who achieved minimal residual disease (MRD)-negative CR after CAR-T cell therapy and underwent allogeneic hematopoietic stem cell transplantation compared to those who did not undergo transplantation. In NHL patients, high levels of serum LDH before lymphodepletion, and low MCP-1 and IL-7 before or shortly after CAR-T cell infusion were associated with shorter progression-free survival after treatment with CD19-CAR-T cells (65).

CD19-CAR-T cells represented salvage therapy also for patients diagnosed with NHL, including advanced DLBCL, primary mediastinal B-cell lymphoma (PMBCL) and follicular lymphoma (FL). Complete responses (CR), 49%–71%, have been observed in these patients, with a median (overall survival) OS greater than 2 years (16, 18, 22, 66, 67). FDA and EMA approval for axicabtagene ciloleucel in relapsed and refractory DLBCL was granted based on results of the multi-center Phase 2 ZUMA-1 trial, presented in a keynote session by Dr. Frederick L. Locke (Moffitt Cancer Center). Lisocabtagene maraleucel (Liso-cel; JCAR017) represents another CAR-T-cell-based therapy for aggressive NHL patients that is composed by 1:1 ratio of both CD4^+^ and CD8^+^ T cells. In the context of the multicenter TRANSCEND-NHL-001 trial, an overall response rate (ORR) of 73% [95% CI, (49, 58, 59, 67–75)] was observed. The CR rate was 53% [95% CI, (47, 48, 50–56, 66, 76–78)] with a median follow-up of 12 months (67). It appears lower frequencies of toxicities may have occurred in the TRANSCEND trial, as well as the JULIET trial that tested tisagenlecleucel in DLBCL; both of these therapeutic products contain the 4-1BBζ costimulatory molecule, in contrast to axi-cel which contains a CD28ζ costimulatory molecule (49, 56, 58, 67). It should be noted that these trials used different toxicity grading systems, so comparisons should be interpreted with caution.

The usage of mathematical and statistical modeling to evaluate the efficacy of CD19-CAR-T cell-based clinical trials, revealed that multiple factors, including the frequency of memory T cells in the circulation, the tumor burden and the inflammatory cytokine profile, can affect the extent and duration of patients’ clinical responses (59). The most frequent toxicities attributed to CAR-T cells are the cytokine release syndrome (CRS) and neurologic events. In the majority of cases they are reversible. CRS can usually be controlled by the administration of tocilizumab (anti-IL-6 mAb) with or without corticosteroids (58, 59, 68). Therapies for neurotoxicity are less well-defined, but usually involve anti-seizure prophylaxis and corticosteroids, with addition of tocilizumab (anti-IL-6 mAb) only if there is concurrent CRS. Patients presenting with severe pro-inflammatory conditions, quantified by serological increase in C-reactive protein and ferritin, before the injection of CAR-T cells appear to be at higher risk for toxicities and might benefit from treatment with immunomodulatory agents before the toxicities become severe (18, 65, 68–70). Safety cohorts of ZUMA-1 tested at early stage and the prophylactic administration of tocilizumab, showed reduced frequency of severe CRS but not neurotoxicity. In this study, the development of neurological toxicity was associated with higher level of CD14^+^ myeloid cells and CD4^+^ CAR-T cells in the cerebrospinal fluid (CSF), again implicating a pro-myeloid inflammatory state associated with CAR T toxicity (65, 68–71). These observations highlight that multiple factors could affect patients’ outcome and the development of toxicities; further studies are required to confirm the role of the association of these parameters as biomarkers predicting the risk to develop toxicities upon CAR-T cell treatment.

## The Hematology/Oncology Landscape in Qatar

According to registry data from 2015, leukemia represents the 5^th^ most common malignancy in Qatar across genders and nationalities. NHL, including DLBCL, is the 4^th^ most common cancer among males and represents the most frequent lymphoid malignancy in Qatar with about 46% of all lymphoid malignancies. The average OS of DLBCL patients has been reported as 64% between 2013 and 2017. However, 30% to 40% of patients has a high risk of relapse from the first line of chemotherapy treatment, and 10% to develop refractory disease, with poor prognosis and a median survival lower than 1 year (72). Dr. Ruba Y. Taha **(**NCCCR-HMC, Qatar) summarized the available therapeutic options for these patients, including the treatment with platinum- and cytarabine-based regimens alone or prior to autologous HSCT (PARMA prospective multicenter study) (73). Patients enrolled in this study with early relapses showed similar clinical outcome as incomplete responders (74). However, these patients have superior clinical benefit from the usage of monoclonal antibodies targeting antigens expressed by blasts (CD19, CD20, CD22, and CD52) as compared to chemotherapy or targeted therapy (54). Therefore, the availability in Qatar of CAR-T cell therapy targeting the aforementioned antigens will provide further options to improve the OS of patients with relapsed/refractory DLBCL. A limited number of patients can benefit from this type of therapy only upon admittance to international clinical centers.

Qatar Cancer Society (QCS) is a charity that was founded in 1997 dedicated to implement the research in this field and orchestrate a community partnership platform to make Qatar among the leading countries in cancer prevention and burden control (Dr. Hadi Mohamad Abu Rasheed, QCS, Qatar). In this context, QCS is facilitating the access of Qatari patients to innovative therapies such as CAR-T cells. A testimonial of a patient with a diagnosis of NHL who underwent a successful CD19-CAR-T cell treatment in US, was presented during the conference, corroborating the need of implementing local efforts and research in this field.

The pediatric cancer genome project showed the differential make-up of driver mutations and related aberrant molecular pathways in these patients as compared to adult cancer patients’ profile (75). These evidences indicate the need to deeply understand the disease’s mechanisms of children with cancer in an attempt to allow early detection and intervention. The comprehensive biological characterization of pediatric leukemia ongoing at Sidra Medicine (Dr. Chiara Cugno, Sidra Medicine, Qatar) is aimed at identifying dysregulated signaling pathways to contribute to the development of precision medicine treatments. These investigations might also lead to identifying novel target molecules for CAR-T cells or the optimization of targeting multiple antigens in relationship with the evolution of the disease and possible development of resistance to this therapy (79, 80).

## Targeting Tumors With TCR Engineered T Lymphocytes

ACT studies with T cells engineered with TA-specific TCR, provided evidences of their clinical success (**Table 2**) (2, 28–31, 34, 81, 82). Nevertheless, the improvement of the efficacy, the tumor specificity and strategies to overcome the competition in the synthesis of TCR between exogenous and endogenous alpha and beta chains are subjects of multiple novel investigations. In addition, the usage in most of the cases of epithelial-derived TAAs, that are shared with normal tissues can induce “off-target” toxic effects. The accurate choice of TAAs selectively expressed by tumor cells and/or by the associated blood vessels and microenvironment can overcome the unwanted unspecific targeting and the related toxicities.

These topics were addressed by the presentations, that are summarized in paragraphs 4 and 5, discussing advanced tools to implement the engineering of T and NKT lymphocytes and refining the selection of TAs to redirect the cell-mediated responses.

Prof. Chiara Bonini, (San Raffaele Scientific Institute, Milan, Italy) presented the application of genetic knock-out (KO) and gene substitution approach to introduce TA-specific TCRs in T lymphocytes. This aim was achieved through the transient exposure of T cells to alpha and beta chain specific Zinc Finger Nucleases (ZFNs), followed by the introduction of lentiviral vectors (LV) encoding for a “novel” TCR (83). This study was focused on targeting the leukemia-associated TA Wilms’ tumor 1 (WT1), that is a zinc finger DNA-binding protein acting as transcription factor and playing an important role in cell growth and differentiation of cells (84). The enrichment of T cell memory stem (TSCM) (85, 86) cells that are “younger” cells with persistence and survival properties and delayed ageing and exhaustion (87) occurred for TA-specific T cells. This population of T cells was found to be enriched in the circulation of patients following HSCT and the infusion with T lymphocytes genetically modified to express the thymidine kinase (TK) suicide gene (88, 89). This phenomenon was observed also following long term treatment with engineered T cells (87), indicating that both antigen exposure and the phenotype of the infused cells can affect their *in vivo* survival and the diversification of their immunological memory (89). ACT can benefit from HSCT platforms followed by the infusion of TCR engineered with gene editing technology in inducing the Graft-versus-tumor (GvT) effect in hematological malignancies (90).

The transfer of lipid specific TCRs into T cells represents another innovative approach for the ACT of leukemia. T cells can recognize lipid antigens presented by MHC class I-related CD1 molecules (CD1a, b, c, d). These T cells are involved in antimicrobial immunity and, in case of reactivity against CD1-presented self-lipids, in autoimmunity and cancer immunosurveillance (91). Dr. Giulia Casorati (San Raffaele Scientific Institute, Milan, Italy) demonstrated that primary acute myelocytic leukemia (AML) leukemia and B-ALL blasts express CD1c and are recognized by a group of CD1c self-reactive T cells specific for methyl-lysophosphatidic acids (mLPAs). mLPAs is a self-lipid antigen highly enriched in malignant cells that might play a role in leukemia growth. Lipid antigens are also less susceptible to mutation and immune mediated selection (92, 93). The anti-tumor activity *in vitro* and the control of the progression *in vivo* of leukemia cells by T cells engineered with mLPA-specific TCRs has been assessed, showing that these T cells could represent novel and efficient tools for ACT for leukemia patients either in the prophylactic setting at the time of HSCT or at post-transplant relapse of the disease (93).

A novel high-throughput technology, XPRESIDENT^®^, has been applied to identify and qualify peptides derived from TAAs (Dr. Ali Mohamed, Immatics, Tubingen, Germany and Houston, TX, USA). This platform, through the combination of ultra-sensitive mass spectrometry (LC-MS/MS) with quantitative transcriptomics, was able to identify the differential expression of HLA-bound peptides in tumor vs. normal tissues. Taking advantage of this platform, several T cell-based therapy programs have entered clinical development. These include the targeting of multiple TAAs with T cells expressing either native or exogeneous TCR. A robust clinical grade manufacturing process has been developed for autologous TCR transduced T cells that implied 7–10 days of manufacturing, (IMA201 and IMA202) or 5–6 days (IMA203), shortening the timeline required for the production in autologous setting. An interesting strategy was based on the engineering of allogeneic γδ T cells, that display inherent anti-tumor activity, the ability to infiltrate tumor tissues and MHC-independent reactivity, with exogenous TA-specific αβ TCR (ACTallo^®^ IMA301). Therefore, these cells acquire αβT cell features in the absence of the risk to develop allogeneic reactions or GvHD due to HLA mismatching (94, 95). Additionally, the development pipeline includes T-cell Engaging Receptors (TCER^®^s). These bispecific TCR molecules are soluble fusion proteins with two binding domains: (i) a TCR domain that recognizes a tumor-specific peptide presented by HLA class I, and (ii) a T-cell recruiting antibody domain, which allows the recruitment and activation of T cells to attack the tumor.

## Optimizing the Delivery *In Vivo* of CAR-T Cells and Their Redirection to Tumors

The gene transfer platforms for therapeutic application can occur either by isolating *ex vivo* the cells for modification and the subsequent re-infusion in the patients or *in vivo* through viral vectors (LV or adenovirus, AAV). The vector delivery can be controlled *via* particle-receptor interactions. The engineering process for specific targeting involves the mutation of the receptor binding sites and adding the desired target domains in these regions (96). Designed ankyrin repeat proteins (DARPins) can recognize target receptors with high specificities and affinities and when ligated into vectors mediate cellular binding (97). Prof. Christian Buchholz (Paul-Ehrlich-Institut, Federal Institute for Vaccines and Biomedicines, Langen, Germany) discussed the selective delivery of CARs into CD8+ T lymphocytes for the *in vivo* generation of CAR-T cells. LVs that can specifically transduce human CD8^+^ cells have been used to deliver the CD19-CAR *in vivo* into immunodeficient mice transplanted with human peripheral blood mononuclear cells (PBMCs). The vector administration in fully humanized mice transplanted with human CD34^+^ cells induced TA-specific CAR-T cells depleting the CD19^+^ B lymphocytes. Signs of CRS, the presence of tissue‐invading CAR-T cells and the complete elimination of the B‐lymphocyte‐rich zones in spleens were detected (98). More recently, efficient eradication of tumors in transplanted mice was demonstrated upon a single vector injection (99). Although further investigations are desirable, these promising results have demonstrated that the *in vivo* engineering of human CD8^+^ CAR-T cells could substantially simplify their manufacturing and the development of future cell-based immunotherapies.

Prof. Soldano Ferrone (Massachusetts General Hospital, Harvard Medical School, Boston MA, USA) has dedicated many efforts to elucidate the immunogenic properties of human chondroitin sulfate proteoglycan (CSPG)-4 and its expression on the membrane of different types of cancer cells, including cancer initiating cells (100–104). This molecule is also known as high molecular weight-melanoma associated antigen (HMW-MAA), or neuron-glial antigen 2 (NG2). CSPGs are key bioactive molecules which play a role in cancer cell growth, migration and neo-angiogenesis (105). This antigen can be upregulated on cancer cells incubated under hypoxic conditions and is not or poorly expressed on normal cells with the exception of pericytes in the TME. Prof. Ferrone’s team has previously isolated the mAb scFv-Fc C21 specifically reacting with CSPG4 (100, 101) and he pointed out that some of the commercially available antibodies, including those used to generate the information about CSPG4 presented in the Atlas of Proteins, have the wrong specificity. This has generated some of the misleading information presented in the Atlas of Proteins. CSPG4 represents a target TA for T cell-mediated responses (101, 105–107). In addition, the availability of specific mAbs for this antigen has allowed the generation of CAR-T cells targeting either epithelial cancers, such as triple negative breast cancer, squamous cell carcinoma of head and neck and glioblastoma (106, 108–110). Interestingly, cancer initiating cells isolated from these types of cancers could be efficiently recognized and killed *in vitro* by CSPG4-CAR-T cells (108, 110). The anti-tumor activity of CAR-T cells targeting CSPG4 was potentiated through the intratumoral injection of effector cells. These pieces of evidence suggest the relevance of CSPG4 as target TA of CAR-T cells.

The development of novel scFvs, through the generation and screening of monoclonal antibodies targeting TAAs, can be applied to redirect engineered T cells for the targeting of colorectal cancer stem-like cells (CRC-SC; Dr. Eleonora Ponterio, Catholic University and Fondazione Policlinico Universitario “A. Gemelli”-I.R.C.C.S. Rome, Italy; Oral poster presentation). This approach represents a novel framework to implement the efficacy of CAR-T cells for solid tumors. Through the injection into mice of primary CRC-SC, antibody-mediated immune responses selectively binding to CRC tissues and not to normal mucosa have been generated. Upon sequencing of the variable chains of these mAbs, the scFv was tagged with a constant fragment (Fc) and used to isolate, through immunoprecipitation, the recognized TAs. The scFvs have been then shuttled into LVs encoding for either 2^nd^ or 3^rd^ generation CARs and used to transduce the immortalized T cell line Jurkat. These cells, upon the activation of the CAR through the co-culture with CRC-SC, secreted IL-2. The isolated mAbs could represent a powerful new tool to direct T cells against CRC [unpublished data and see (111)].

The deep characterization of the mechanisms for differences in cancer phenotypes could contribute to understand patient’s clinical outcome and the responsiveness to immunotherapy. A pan-cancer analysis of the TCGA dataset has revealed that a pre-existing intratumoral T helper (Th-1) immune response can affect the OS of cancer patients with variable outcome depending on the histological origin of tumor. This indicates that cancer-specific pathways modulate the prognostic power of anti-tumor immune response and shape the TME and the type of immune infiltrating cells (Jessica Roelands, PhD Student, Sidra 805 Medicine, Qatar; Oral poster presentation) (112–115). These observations can play a role in the stratification of cancer patients. The clinical relevance of these findings was demonstrated using a dataset of melanoma patients treated with checkpoint inhibitors. A high expression of genes reflecting Th-1 anti-tumor responses in pretreatment samples was associated with improved survival only for samples with high proliferation scores, or with low TGF-β expression (115). The investigation of these parameters in tissues form large cohorts (N=366) of CRC patients represents a valuable tool to either predict the responsiveness of patients to immunotherapy or to identify novel target molecules for immune-based therapies.

## Manufacturing Implementation of Engineered T Cells and Pharma/Biotech Perspectives

The manufacturing of CAR-T cells over the time underwent a rapid evolution, from the 1^st^ until the 3^rd^ generation of CAR-T cells, depending on the structure and number of co-stimulatory molecules included in the signaling portion of the receptor. The choice of 2nd generation CAR-T cell for therapeutic application resulted from the practical need to offset toxicity rather than to achieve greater anti-tumor efficacy. Further advances are the “armored” CAR-T cells. These represent genetically modified T cells bearing CARs that comprise in their structures immunomodulating agents such as: i. proinflammatory cytokines; ii. secreted antibodies or their part bearing antigen specificity; iii. costimulatory ligands (Dr. Renier Brentjens, Memorial Sloan Kettering Cancer Center, USA). Armored CD19-CAR-T cells bearing the murine IL-18 (mIL-18 CAR-T) showed in *in vivo* models enhanced expansion and persistence, anti-tumor activity and prolonged B cell aplasia (up to 150 days after treatment), dependent on autocrine IL-18R signaling (116), even without prior chemotherapy conditioning. Whereas, without preconditioning of mice, the anti-CD19 CAR-T cells failed to prolong the survival of animals in a syngeneic tumor model (117). Through targeted delivery of IL-18 to the tumor, mIL-18 CAR-T cells can modulate the tumor microenvironment, recruit, and activate endogenous anti-tumor immune effector cells, which in turn orchestrate an effective anti-tumor response beyond the CAR-specific targets (116). Programmed cell death-1 (PD-1)/ligand (PD-L1) signaling represents one of the immune checkpoints that can modulate CAR-T cell-mediated responses when they encounter either tumor cells or TME. Armored CAR-T cells endowed with the capacity to secrete anti-PD-L1 scFv showed enhanced in vivo anti-tumor activity, persistence and survival in a PD-L1^+^ tumor model. PD-1-blocking Ab produced by CAR-T cells can also enhance the activity of tumor-specific “bystander” T cells present in TME. Unlike the usage of systemic checkpoint blockade therapy with monoclonal antibodies, the scFv secreted by CAR-T cells penetrate and remain in the TME improving their anti-tumor activity (118). Moreover, the safety of these armored CAR-T cells is superior than checkpoint blockade systemic therapy (118). The third example of armored CAR-T cells comprise the immunostimulatory CD40 ligand (CD40L), that is a type II transmembrane protein belonging to the TNF receptor family. CD40L expressing CAR-T cells displayed enhanced proliferation and secretion of proinflammatory cytokines, e.g., IFN-γ and GM-CSF (119). These armored effector T cells demonstrated enhanced cytotoxic activity both *in vitro* and *in vivo* for both CLL and NHL models (119, 120). All together, these evidences, indicate the promising clinical efficacy of next generation armored CAR-T cells.

The clinical grade manufacturing of CAR-T cells can be challenging due to the complexity of the process. The collaboration between biotech and researchers in academia can result in the development of novel technological platforms to support researchers and physicians from discovery to the clinical development of cell therapy (64, 121). One example is represented by the automated closed system production of engineered T cells that can perform all phases of CAR-T cell generation, from magnetic T-cell enrichment, activation, viral transduction and expansion to downstream harvest for cryopreservation/infusion (CliniMACS Prodigy system). The progress in the generation of this type of system allow standardization of processes facilitating transfer between different manufacturing facilities while the flexibility of the manufacturing platform supports the rapid transfer of innovative cell therapies to the clinic (Dr. Ian Johnston, Miltenyi Biotec B.V. & Co. KG, Germany).

## Genetic Engineering of Cells and Gene Editing

Genetic engineering can act on the core functioning of cells, including reprogramming, differentiation, migration, fitness, and proliferation. In addition, gene therapy is aimed at correcting genetic and degenerative diseases. Novel tools have been developed for the precise engineering of immune cells.

Retroviral vectors (RVs), one of the most effective approaches to the modification of genes, display the ability to integrate at various positions in the genome. This implies the risk of mutagenesis in the course of the integration of vectors into the genome. Studies on animal models have revealed evidence of leukemia induction in a process of insertional mutagenesis mediated by retroviral vector (122). The utilization of constructs with improved safety, such as the low-risk recombinant alpharetroviral SIN vectors (aRV) can prevent the genotoxicity caused by insertional mutagenesis (Prof. Axel Schambach, Hannover Medical School, Germany), as SIN-aRV exhibit a neutral integration pattern, lowering the risk of insertional mutagenesis as compared to gammaretroviral and LV (123, 124). Given that aRV vectors do not have the tendency to integrate their cargo into promoter/enhancer regions as well as gene bodies, insertional damages arising from the integration of aRV are lower as compared with other RVs. LVs are considered relatively safe due to their tendency to insert into genes instead to the transcriptional sites that may lead to insertional mutagenesis. The usage of transient retroviral platform, such as the retroviral episome transfer (RET), allows a controllable transgene expression, low or absence of cytotoxicity and the option for cell targeting (123, 124).

Genome editing tools like CRISPR/Cas9, transcription activator-like effector nuclease (TALENs), zinc fingers nuclease (ZFNs), Mega-tal and HEs provide a precise method for the genetic engineering of cells. These platforms introduce double strand breaks (DSBs) in the genome that can be repaired either by non-homologous end-joining (NHEJ) causing disruption of the genes or by homologous recombination which happens at lower rates allowing nucleotide modification of the genome. CRISPR/CAS9 platform requires the co-delivery of a single guide RNA (sgRNA), representing the bottleneck of this strategy. The MS2 bacteriophage RNA packaging mechanism has been applied to the production of non-integrating RVs, GV.MS2-CRISPR/Cas9 all-in-one particles, for efficient gene editing (125, 126). This strategy led to efficient KO of target genes, such as TP53 in human fibroblasts or CXCR4 or CCR7 in Jurkat or T cells, respectively (125–127). These platforms are applicable to the generation of allogeneic CAR-T cells to prevent GvHD and/or to engineer their immune functions.

The usage of ribonucleoproteins (RNPs) as the primary mode of gene editing variants of high-fidelity Cas9 (IDT HiFi SpCas9) enabled better gene-editing by reducing levels off-target INDELs as identified by bioinformatics tools (e.g., COSMID, guide-seq genome-wide profiling of target cells or circle-seq *in vitro* screen for genome-wide CRISPR/Cas9 nuclease off target) (Prof. Matthew Porteus, Stanford University, San Francisco, CA, USA) (128). This represents an efficient tool for CD19-CAR-T cell to lower the associated toxicities and implement the anti-tumor activity. CD19-CAR-T cells employed with the KO of TCRab to avoid GvHD were assessed in the context of infusions before and following TCRab-depleted haploidentical-HSCT (129). This was achieved through the targeting of the locus of TCR (TRAC) with CRISPR/CAS9 non-integrating AAV6 encoding forCD19-CARs. The engineered T cells showed active anti-tumor activity against CD19^+^ leukemia xenografts (Nalm6 cells in NSG mice). No off-targets indels was detected in these CAR-T cells through the usage of HiFi Cas9 (129). In order to improve further CAR-T cell efficacy and safety, in 2017, Dr Eyquem et al. (130) used complex genome editing tool to demonstrate that TCR-like expression of the CAR improve CAR-T therapeutic activity. However, genome editing tool to demonstrate that TCR-like expression of the CAR improves CAR-T therapeutic activity. In this direction, Prof. Francisco Martin (Pfizer-University of Granada-Junta de Andalucía Centre for Genomics and Oncological Research, GENYO, Spain) presented an interesting approach to facilitate clinic translation of TCR-like CAR-T cells. Dr. Martin´s team constructed LVs that closely followed the expression profile of the TCR upon CD3 stimulation and generated CAR-T cells using the LVs (*Patent PCT/EP2019/081346, “Polynucleotide for safer and more effective immunotherapy”* and unpublished data). These physiological CAR-T cells showed strong resistance to exhaustion upon repeated stimulation and showed potent *in vivo* anti-tumor activity. The author proposes the use of these physiological LVs (similar to the one already approved by FDA) to translate TCR-like CAR-T cells into clinic. Another important aspect to improve the potency and safety of CAR-T cells stands on the design of new tools that allow the clinicians to be able to externally control their activity. Dr. Martin presented their latest development on their transactivator-independent doxycycline-regulated LVs (Lent-On-Plus). They showed the potency of this system to induce any transgene in T cells both *in vitro* and *in vivo* with very low doses of doxycycline. As proof of concept, he also presented the ability to generate inducible CAR-T cells that selectively kill CD19^+^ cells only in the presence of doxycycline. However, the author envision that this approach will be more applicable for future development of inducible TRUCKs (iTRCKs), expressing any desired cytokine (factor) upon the addition of doxycycline.

## Allogeneic/”Off-the-Shelf” Therapy With CAR-T Cells

The generation of autologous CAR-T cells for therapeutic treatment implies many challenges related to i. the isolation of circulating lymphocytes from heavy pre-treated patients who might have lymphopenia; ii. the manufacturing of the cells; iii. the logistics of collection of the starting material, preparation and delivery for infusion of the medicinal product into patients; iv the inter-patient variability in the yield and quality of the cell product. The major advantage of generating universal CAR-T cells is their “off-the-shelf availability, without delay in the treatment and suitability for multiple infusions of engineered T cells. Moreover, the manufacturing of universal CAR-T cells allows the implementation of the standardization of batches of cells and to reduce the costs of production. The strategy to generate anti-tumor CAR-T cells starting from peripheral blood lymphocytes isolated from healthy donors, has also the potential to overcome the impairment of effector functions of patient-derived CAR-T cells.

Universal CD19-CAR-T cells (UCART19) represent the first “off-the-shelf” CAR-T cell product targeting CD19 expressing malignancies (Dr. Reuben Benjamin, King’s College Hospital, UK). In this product, anti-CD19 scFv-41BB-CD3ζ CAR is expressed in T cells, with KO of TRAC and CD52 genes to prevent GvHD in HLA mismatched patients. The TRAC and CD52 gene KO has been performed through the usage of TALEN (41). In addition, RQR8 epitope, that is a mimotope of CD20 has been introduced into engineered T cells as a safety switch (131). The safety and tolerability of UCART19 in pediatric and adult patients with diagnosis of ALL have been tested in the context of the PALL/CALM study following lymphodepleting regimens and to evaluate the maximum tolerated dose (MTD). The study revealed that UCART19 has a safety profile, with no acute GvHD and severe toxicities, leading to 67% of CR (40, 43, 132).

Different strategies of gene editing are in place for allogeneic CAR-T cells for the knock-out of genes involved in either GvHD, such as TCRαβ - TRAC, TCRBC1, TCRBC2, rejection molecules (e.g., CD52, β2 microglobulin), or molecules associated with the exhaustion of T cells (e.g., PD-1, CTLA-4) (42, 44). CAR-T cell approaches in infant ALL patients have sometimes encountered difficulties in generating autologous CAR-T cells and, moreover, are associated with the risk of developing CD19-tumor cell escape (Prof. Paul Veys, Great Ormond Street Hospital For Children & UCL GOS Institute of Child Health, UK). UCART19 represented a bridge between lymphodepletion/CAR-T cell therapy and allogeneic HSCT. This strategy allowed the eradication of CD19^-^ malignant cells following CAR-T cell therapy during the allogeneic HSCT, followed by reconstitution of donor immune cells and elimination of residual UCART cells. This procedure was followed by a 30-day transient GCSF-dependent recovery of neutrophils and protracted multilineage cytopenia until the second allo-SCT after 12 weeks. The TALEN trial achieved 60% clinical responses in pediatric ALL patients. The effectiveness of TALEN versus CRISPR/Cas9 was compared, indicating that the risk of GvHD might be reduced with CRISPR/Cas9, as CAR insertion and editing of TCR were coupled, leading to reduced potential contamination of the final product with TCR positive cells. The generation of CAR-T cells from umbilical cord blood (UCB) coupled with gene editing has been explored in pre-clinical models as alternative method to generate allogeneic CAR-T cells with increased early differentiation features, including increased proliferative capacity (133). The unmet need is to define the optimal lymphodepletion regimen(s) and whether allo-SCT is required post UCART infusion to extend the clinical benefit of this cell-based therapy.

The usage of UCB as starting material to generate CAR-T cells represented the object of one study ongoing at Sidra Medicine (Dr. Cristina Maccalli, Sidra Medicine, Qatar). UCB offers the unique capacity of broad HLA mismatch between donor and host. The frequency of T lymphocytes in cord blood is lower as compared to peripheral blood representing a limiting factor for the generation of CAR-T cells. The CD19-CD28ζ and CD19- 4-1BBζ CAR-T cells have been isolated and phenotypically characterized utilizing an in-house designed multiparametric IF (including 28 markers) panels. The overall phenotype at different time points (Day 0, + 7, +9, +14) of *in vitro* culture of UCB vs. peripheral blood-CAR-T cells varied in terms of the percentage of CD4^+^ and CD8^+^ stem/central memory T cells. In addition, immunomodulating molecules such as PD-1, TIM3, and LAG3 were differentially expressed in CAR-T cells with different origins. The integration of different platforms, such a flow cytometry, EliSpot, and FluoroSpot based cytokine release and transcriptomic profiling (134) allowed the development of a proof of principle study aimed at identifying the UCB-derived CAR-T cell population endowed with superior anti-tumor activity. Further investigations are warranted to validate these results (unpublished data).

CAR-NK cells represent a promising strategy for allogeneic and universal cell-therapy (Dr. Tomas Bos, Glycostem Therapeutics, The Netherlands). NK cells represent the 5%–10% of circulating immune cells and are members of innate immune control with low risk, due to their independence on the recognition of MHC/peptide complexes, of allogeneic rejections or GvHD upon their infusion in cancer patients. The generation of clinical grade NK cells has been optimized in the presence of a liquid cell culture containing differentiation factors and an artificial niche composed of glycosaminoglycans. The NK cell product, denominated oNKord^®^, showed strong cytotoxicity against CRC, regardless of the mutational status of EGFR and RAS (135), and leukemia both *in vitro* and in mouse models (136). Interestingly, this type of treatment in a phase I clinical trial for AML patients following conditioning with Flu/Cy, showed safety, no toxicities or sign of GvHD and clinical efficacy with improvement of survival (34 months) (137).

The repertoire of available gene editing tools for CAR-T cells has been discussed by Dr. Karim Benabdellah **(**GENYO, Spain; Oral Abstract Presentation), highlighting that the insertional deficient LV episomes (IDLV) combined with CRISPR/CAS-9 can implement the effciency of CD19-CAR-T cells for the KO of TCR to prevent *in vivo* allogeneic rejection and GvHD. These CAR-T cells can maintain both *in vitro* and *in vivo* the reactivity against tumor cells.

## The Usage Of maB N Cancer Therapy As A Complementary Or Integrative Approach For Engineered Immune Cells

Several mAbs have been registered as standard of care for different types of tumors. Some of them, through the binding to the Fc*γ* receptor (Fc*γ*R) on effector cells, such as NK and *γ*δ T cells, can activate the antibody-dependent cell-mediated cytotoxicity (ADCC) that can lead to the killing of malignant cells (Prof. Hans van der Vliet, Amsterdam University Medical Center, Cancer Center Amsterdam, The Netherlands). Bispecific Ab, bearing two epitope binding regions, have been designed to bind simultaneously to two target molecules and can be used to target immune effector cells to tumor cells. Trifunctional hybrid antibodies (Triomabs) are formed by combining two halves of distinct mAbs, for example of rat and mouse mAb origin, which, as a result of species preferential heavy-light chain pairing, reduces random pairing of heavy and light chains simplifying manufacturing. In addition, to facilitate the interaction of immune cells with target cells, Triomabs (e.g., catumaxomab, ertumaxomab) can also mediate ADCC through the Fc*γ*R (138–141). Notably, other bispecific antibody formats utilize a linking of single chain Fv (scFv) fragments with different antigen specificity, for which several formats can be used. An example of a scFv based bispecific antibody in a tandem orientation (TaFv) is blinatumomab, an anti-CD3 and anti-CD19 bispecific T cell engager (BiTE) used for the treatment of, e.g., relapsed or refractory B-ALL, that showed an improvement of complete remission rate and of OS as compared with chemotherapy (53, 142). Multiple alternative bispecific antibody platforms (e.g., bispecific diabodies, Fc-containing bispecific antibodies, immunocytokines, prodrug bispecifics, and MHC class I targeting bispecifics) are under development in an effort to further improve the clinical efficacy of such approaches and to extend their application towards other disease indications (143–145). The generation of novel antigen-specific scFv could also complement the function of CARs, either by enhancing the binding to target molecules or expanding the tools to redirect CAR-T cells to tumor sites. In addition, the usage of mAbs, including the bispecific, either in combination with CAR-T cells or encoded simultaneously with CARs by LV vectors, can represent an interesting approach to enhance the homing of CAR-T cells to the tumor site, their penetration into the TME and the anti-tumor activity (e.g., armored CAR-T cells discussed in paragraph 6).

## Conclusions

The development of engineered immune cells, T lymphocytes and NK cells, represents a breakthrough of cancer immunotherapy for both blood and solid tumors (see **Tables 1** and **2**). For the first time, one of these innovative therapeutic strategies (CAR-T cells) has been approved as standard of care for some hematological malignancies. Nevertheless, the optimization of these interventions, in terms of identification of novel TAs, the improvement of the survival and the limitation of differentiation, aging and exhaustion of T cells, as well as of associated toxicities upon *in vivo* infusion and their capacity to penetrate TME are under investigation. Several platforms and approaches have been developed to mitigate the aforementioned limitations of anti-tumor cell-based therapies, contributing to the rapid evolution of the field. Furthermore, novel Ab-based tools designed on tumor cell phenotype and cancer immune setpoints are warranted to design more effective personalized Ab-based weapons for cancer therapy. The development of these approaches has required long path for R&D, however, the gained expertise could facilitate the implementation of the molecular and genomic tools to generate anti-tumor cell therapy with superior clinical efficacy. Nevertheless, the discussion occurring in the context of the conference highlighted the need of specialized manufacturing facilities and clinical centers, including multidisciplinary personnel, for the production, delivery and infusion into patients, as well as their clinical monitoring and follow-up. The development of inter- and intra-regional networks is of high relevance to facilitate the establishment of these cell-therapy centers and to expand the accessibilities to these therapeutic interventions to a broad number of cancer patients. The costs associated with the development of this type of centers and with the clinical grade manufacturing of these cellular medicinal products should also be taken into account. The access of patients to these therapies and their attractiveness for clinical application might increase whether a point-of-care manufacturing model would be designed and established. The role of pharma and biotech has been shown to be critical and to facilitate the clinical grade development of these approaches. The interaction and cooperation between academia and industry were emphasized during the sessions’ discussions as in need of being potentiated and initiated even at early phase of R&D. Further investigations are also required to simplify the manufacturing of engineered immune cells and potentiate the development and safety of allogeneic products. The exploration of the combination therapies (such as with immunomodulating agents and/or standard therapies) could represent a strategy to overcome the development of resistance to cell-based therapies and to target a broad variety of tumors. A consensus regarding the need of continuous exchange of information among investigators and clinicians and of the development of international networks and collaborations resulted from the participants to the workshop.

## The EICCI Faculty Group

Karim Benabdellah, Pfizer/University of Granada/Andalusian Regional Government, Genomic Medicine Department, Granada, Spain; Tomas Bos, Glycostem Therapeutics, AB Oss, The Netherlands; Chiara Cugno, Research Department, Sidra Medicine, Doha, Qatar; Ian Johnston, R&D, Miltenyi Biotec, Bergisch Gladbach, Germany; Ali Mohamed, Immatics Biotechnologies, Tubingen, Germany; Eleonora Ponterio, Catholic University and Fondazione Policlinico Universitario "A. Gemelli"-I.R.C.C.S. Rome, Italy; Hadi Mohamad Abu Rasheed, Qatar Cancer Society, Doha, Qatar; Jessica Roelands, Research Department, Sidra Medicine, Doha, Qatar; Ruba Y. Taha, Hematology-BMT Department, National Cancer Center for Care and Research (NCCCR), Hamad Medical Corporation, Doha, Qatar.

## Author Contributions

BG, ME, AA-M, and DK prepared the draft of the manuscript and the final editing. CM contributed to the preparation, revision, and editing of the manuscript. RBe, CBo, RBr, CBu, GC, SF, IJ, AM, FL, FM, AS, CT, PV, and HV provided critical inputs and revised the manuscript. KBL, TB, EP, JR, HMAR, and RYT contributed to the editing of the text. All authors contributed to the article and approved the submitted version.

## Funding

The EICCI workshop was funded by the Qatar National Research Fund (QNRF)- Conference Workshop Sponsorship Program grant CWSP15- W-0911-18001 (Doha, Qatar). CBo received funding from AIRC (Ig 18458) and AIRC 5 per Mille, Rif. 22737, Italian Ministry of Research and University (PRIN 2017WC8499) and Italian Ministry of Health (Research project on CAR T cells for hematological malignancies and solid tumors).

## Conflict of Interest

CBo received a research contract from Intellia Therapeutics and participated to the advisory boards of Molmed, Intellia Therapeutics, TxCell, Novartis, GSK, Allogene, Kiadis. CBu is the inventor of patents in the field of adoptive T cell therapy. RB is a recipient of research funding from Servier. IJ is employed at Miltenyi Biotec. FL has scientific advisory role for Kite, a Gilead Company, Novartis, Celgene/Bristol-Myers Squibb, GammaDelta Therapeutics, Wugen, Amgen, Calibr, Amgen, and Allogene; is a consultant with grant options for Cellular Biomedicine Group, Inc.; and has research support from Kite, a Gilead Company. AM is employed at Immatics. CT received research funding from Juno Therapeutics/BMS, Nektar Therapeutics, Minerva, AstraZeneca, and TCR^2^ Therapeutics. He is a member of Scientific Advisory Boards of Precision Biosciences, Eureka Therapeutics, Caribou Biosciences, T-CURX, Myeloid Therapeutics, ArsenalBio, and Century Therapeutics, and *ad hoc* advisory boards (last 12 months) of Nektar Therapeutics, Allogene, PACT Pharma, Astra Zeneca, and Amgen. He has stock/options of Precision Biosciences, Eureka Therapeutics, Caribou Biosciences, Myeloid Therapeutics, and ArsenalBio. CT is the inventor of a patent licensed to Juno Therapeutics. PV is a recipient of a grant from SERVIER to investigate Universal chimeric antigen receptor T cells for ALL (UCAR19-PALL). HV is employed as a chief scientific officer (CSO) of Lava Therapeutics.

The remaining authors declare that the research was conducted in the absence of any commercial or financial relationships that could be construed as a potential conflict of interest.

## Abbreviations

AAV, Adeno-Associated Virus; ACT, Adoptive Cell Therapy; ADCC, Antibody-Dependent Cell-Mediated Cytotoxicity; BMT, Bone Marrow Transplant; CAR, Chimeric Antigen Receptor; CCR7, C-C Chemokine Receptor Type 7; CD1 a, b, c, d, family of glycoproteins expressed on the surface of various human antigen-presenting cells and related to HLA class I molecules; CD19, IgSF surface glycoprotein of 95 kDa expressed on B cells; CD20, B-lymphocyte antigen CD20; CD22, B-lymphocyte cell adhesion molecule; Sialic acid-binding Ig-like lectin 2 (SIGLEC-2); CD28, Cluster of Differentiation 28, T-cell-specific surface glycoprotein; CD40/CD40L, Cluster of differentiation 40/Ligand of CD40; CD52, Campath-1 antigen; CD54, Cluster of Differentiation 54 or Intercellular Adhesion Molecule 1 (ICAM-1); CD58, Lymphocyte function-associated antigen 3 (LFA-3) CD70, Cluster of Differentiation 70; CXCR4, C-X-C Chemokine Receptor Type 4; CD80, Cluster of differentiation 80 (also B7-1); CD86, Cluster of differentiation 86 (also B7-2); Cy, Cyclophosphamide; COSMID, Database of Genomic Structural Variation; CRC, Colorectal Cancer; CRISPR/Cas9, (Clustered Regularly Interspaced Short Palindromic Repeats) and CRISPR-associated (Cas9); CTLA-4, Cytotoxic T lymphocyte Antigen 4; EGFR, Epidermal Growth Factor, Fas, Death Receptor that regulate apoptosis; Fc*γ*R, Fc Gamma Receptor; Flu, Fludarabine; GMP, Good Manufacturing Practice; GvHD, Graft versus Host Disease; HLA, Human Leukocyte Antigen; HSCT, Allogenic Hematopoietic Stem Cell Transplant; IL-6, Interleukin 6; IL-7, Interleukin 7; IL-18, Interleukin 18; KO, Knock out; LAG-3, Lymphocyte Activation Gene-3; LDH, Lactate Dehydrogenase; LV, Lentiviral vector; MCP-1, Monocyte Chemoattractant Protein-1; MEGATAL, Meganucleases that have been fused with a Transcription Activator-Like (TA) containing Repeat Variable Residues (RVD); NK, Natural Killer; OS, Overall Survival; PD-/PD-L1, Programmed Cell Death-1/Ligand; RAS, Rat Sarcoma; RV, Retroviral vectors; scFV, Single-Chain Variable Fragment; TAA, Tumor-Associated Antigen; TCGA, The Cancer Genome Atlas; Tp53, Tumor Protein 53; TSCM, T Cell Memory Stem; TCR, T Cell Receptor; TCRαβ, T Cell Receptor alpha beta; TME, Tumor Microenvironment; TRAC, T-cell receptor α constant locus; TRBC1, T Cell Receptor Beta Constant 1; TRBC2, T Cell Receptor Beta Constant 2; TIM3, T cell immunoglobulin and mucin domain- containing protein 3; UCAR, Universal Chimeric Antigen Receptor; UCB, Umbilical Cord Blood; 4-1BB, activation-induced costimulatory molecule (CD137).
